# Improved Cervical Lymph Node Characterization among Patients with Head and Neck Squamous Cell Carcinoma Using MR Texture Analysis Compared to Traditional FDG-PET/MR Features Alone

**DOI:** 10.3390/diagnostics14010071

**Published:** 2023-12-28

**Authors:** Eric K. van Staalduinen, Robert Matthews, Adam Khan, Isha Punn, Renee F. Cattell, Haifang Li, Ana Franceschi, Ghassan J. Samara, Lukasz Czerwonka, Lev Bangiyev, Tim Q. Duong

**Affiliations:** 1 Albert Einstein College of Medicine and Montefiore Medical Center, Department of Radiology, Bronx, NY 10467, USA; 2 Stony Brook Medicine, Department of Radiology, Stony Brook, NY 11794, USAanna.franceschi@stonybrookmedicine.edu (A.F.); lev.bangiyev@stonybrookmedicine.edu (L.B.)

**Keywords:** texture analysis, cervical lymphadenopathy, PET-MRI, squamous cell carcinoma, machine learning

## Abstract

Accurate differentiation of benign and malignant cervical lymph nodes is important for prognosis and treatment planning in patients with head and neck squamous cell carcinoma. We evaluated the diagnostic performance of magnetic resonance image (MRI) texture analysis and traditional 18F-deoxyglucose positron emission tomography (FDG-PET) features. This retrospective study included 21 patients with head and neck squamous cell carcinoma. We used texture analysis of MRI and FDG-PET features to evaluate 109 histologically confirmed cervical lymph nodes (41 metastatic, 68 benign). Predictive models were evaluated using area under the curve (AUC). Significant differences were observed between benign and malignant cervical lymph nodes for 36 of 41 texture features (*p* < 0.05). A combination of 22 MRI texture features discriminated benign and malignant nodal disease with AUC, sensitivity, and specificity of 0.952, 92.7%, and 86.7%, which was comparable to maximum short-axis diameter, lymph node morphology, and maximum standard uptake value (SUVmax). The addition of MRI texture features to traditional FDG-PET features differentiated these groups with the greatest AUC, sensitivity, and specificity (0.989, 97.5%, and 94.1%). The addition of the MRI texture feature to lymph node morphology improved nodal assessment specificity from 70.6% to 88.2% among FDG-PET indeterminate lymph nodes. Texture features are useful for differentiating benign and malignant cervical lymph nodes in patients with head and neck squamous cell carcinoma. Lymph node morphology and SUVmax remain accurate tools. Specificity is improved by the addition of MRI texture features among FDG-PET indeterminate lymph nodes. This approach is useful for differentiating benign and malignant cervical lymph nodes.

## 1. Introduction

Head and neck cancer accounts for about 4% of all cancers in the United States. When evaluating patients with head and neck cancer, it is important to consider the prognostic and therapeutic implications of nodal metastases. In patients with head and neck squamous cell carcinoma (HNSCC), a single lymph node metastasis results in a 5-year survival rate of 50%, while an additional contralateral nodal metastasis reduces survival to 33% [1]. Since prognosis is highly associated with the presence or absence of nodal disease, accurate assessment is imperative for treatment planning.

The radiologic evaluation for malignant lymphadenopathy is challenging, as multiple imaging features are used to distinguish pathologic from normal or reactive lymph nodes. Common features include lymph node size, morphology, contour, internal heterogeneity, and maximum standardized uptake value (SUVmax) [2,3]. Nodal size, morphology, and SUVmax are some of the more clinically relevant parameters, although size often lacks sensitivity, and SUVmax can be misleading, especially in subcentimeter lymph nodes [3,4].

Medical imaging has evolved into a pivotal diagnostic tool, offering clinicians detailed insights into anatomical structures and pathological conditions. Beyond the visual representations of tissues and organs, the microscopic intricacies within these images hold invaluable information often concealed from the naked eye. Texture analysis pathologies [5,6] emerge as a sophisticated computational approach, venturing beyond conventional image interpretation to decode the intricate patterns and nuances embedded within medical images. Texture, an amalgamation of spatial variations in pixel intensities, delineates the complex interplay of tissues’ microarchitecture, providing a deeper understanding of their composition and organization. Texture analysis delves into this inherent complexity, quantifying and characterizing these subtle variations. It scrutinizes patterns, contrasts, and spatial relationships among pixels, unveiling hidden information pertaining to tissue homogeneity, heterogeneity, and structural nuances. Numerous studies have shown that texture analysis can be helpful for predicting various endpoints, including patient prognosis, response to treatment, and even tumor molecular features [7,8,9,10,11].

Several studies have previously evaluated the use of texture analysis for characterizing malignant cervical lymphadenopathy in patients with HNSCC [12,13,14,15,16,17]. Park et al. [12] used multi-shot EPI-based DWI to distinguish benign and malignant cervical lymphadenopathy by applying texture analysis to the ADC data. Forghani et al. [13] and Seidler et al. [14] also recently showed how dual-energy CT can be used with machine learning for the characterization and evaluation of nodal status in patients with HNSCC. Additionally, Kuno et al. [15] used CT texture features from contrast-enhanced FDG-PET/CT to distinguish nodal metastases from disease-specific nodal reactivity in HIV-positive patients with HNSCC. MRI texture features have been used to predict extracapsular nodal spread in patients with oral cavity cancer [16] and response to chemo-radiotherapy [17].

The aim of this study is to evaluate the use of magnetic resonance texture analysis (MRTA) for distinguishing benign and malignant cervical lymph nodes in patients with HNSCC undergoing FDG-PET/MR imaging. Our study combined traditional imaging features derived from hybrid PET/MR technology, such as lymph node size, morphology, and SUVmax with MRTA to improve nodal assessment.

## 2. Materials and Methods

*Patient Population:* The requirement for informed consent was waived in this institutional review board-approved retrospective study. A search of the Stony Brook Medicine database for medical records from September 2016 to February 2020 was performed to find patients with HNSCC who had undergone pre-treatment evaluation with FDG-PET/MR imaging. Inclusion criteria were (a) age of 18 years or older, (b) diagnosis of histologically confirmed HNSCC, (c) pre-treatment FDG-PET/MR imaging of the neck, (d) histologically confirmed or unequivocally metastatic cervical lymphadenopathy, (e) no prior head and neck cancer treatment, (f) adequate image quality. Twenty-one patients met the criteria. There are no exclusion criteria.

*Imaging Protocol:* All patients were imaged using a dedicated head and neck PET/MR protocol. The patients fasted for 6 h prior to obtaining approximately 9 mCi (333 MBq) of FDG intravenously with weight-adjusted dose modification. All patients had serum glucose levels of 140 mg/dL or lower. PET and MRI were acquired simultaneously, with dedicated neck and body imaging at 60 and 90 min. Intravenous gadolinium-based gadobutrol was given during the neck sequences at 0.1 mmol/kg.

All images were acquired on a dedicated PET/MR hybrid camera (Biograph mMRI, Siemens Healthcare). The MR unit was equipped with a 3 Tesla magnet. PET and MRI data were acquired using a 12-channel head matrix frequency coil for the neck images and a body frequency coil for body imaging. For MR attenuation correction maps, a dual-echo T1-weighted gradient-recalled echo sequence was performed based on a Dixon segmentation. For the PET neck images, the matrix size was 344 × 344 × 127 with a transaxial FOV of 59.4 × 59.4 cm and an axial FOV of 25.8 × 25.8 cm. The voxel size was 1.39 × 1.39 × 16 mm with a 2 mm slice thickness.

Dedicated neck MR sequences included axial and coronal STIR [TR 3200 ms, TE 37 ms, TI 220 ms, slice thickness 4.0 mm, matrix 320 × 320], axial T2-weighted turbo spin echo, coronal 3D T1-weighted SPACE, and pre- and post-contrast axial 3D T1-weighted radial volumetric interpolated breath-hold sequence (VIBE) with fat suppression. Whole body imaging from the top of the head to mid-thigh included axial T2-weighted HASTE, axial T1-weighted radial VIBE, and sagittal T1-weighted turbo spin echo Dixon with fat suppression sequences.

*Image and Texture Analysis:* Each patient’s FDG-PET/MR images were independently reviewed by an experienced neuroradiologist (L.B.) and an experienced nuclear medicine physician (R.M.) who were blinded to the clinical findings and histopathologic results. The maximum short-axis diameter and morphology of all measurable lymph nodes were determined by each reviewer and recorded by laterality and nodal level. Lymph node morphology was considered abnormal by visualization of either a rounded shape, central necrosis, or loss of the normal fatty hilum. The SUVmax values of all measured lymph nodes were calculated and recorded after free-hand volume-of-interest segmentation using the attenuation-corrected images (version 6.5, MIM Software, Cleveland, OH, USA).

Texture analysis of all recorded lymph nodes was then performed using LIFEx software (version 3.74, www.lifexsoft.org, Property of CEA). This was accomplished by manually segmenting regions of interest (ROIs) along the cortex of all measurable lymph nodes visualized on at least two consecutive axial STIR images. Areas of necrosis and normal fatty hila were excluded from the segmented ROIs, as our goal was to evaluate the texture of non-necrotic nodal parenchyma. Lymph nodes smaller than 64 voxels were excluded from texture analysis, as the software is unable to calculate second-order features using less than 64 voxels. A total of 41 texture features were extracted, including 9 first-order parameters, 7 texture features from the Gray-Level Co-occurrence Matrix (GLCM), 11 texture features from the Gray-Level Run-Length Matrix (GLRLM), 3 texture features from the Neighborhood Gray-Level Different Matrix (NGLDM), and 11 texture features from the Gray-Level Zone-Length Matrix (GLZLM).

After texture analysis was completed, we carefully correlated each patient’s recorded lymph nodes by laterality and nodal level with surgical reports and histopathologic analysis to establish our ground truth of benign and malignant lymph nodes. Lymph nodes that could not be correlated with histology were excluded to maintain accuracy.

*Statistical Analysis:* The variables were presented as mean ± standard deviations. Lymph nodes were classified as malignant if they had confirmed histology or if they demonstrated abnormal morphology and SUVmax > 8 (thus termed unequivocally metastatic) in the absence of histologic confirmation. Texture features were compared between benign and malignant lymph nodes using the unpaired Student’s *t*-test or chi square test, as appropriate. Traditional imaging features, including maximum short-axis diameter, morphology, and SUVmax, were also compared between groups.

To determine whether texture and traditional imaging features could predict ground truth in these lymph nodes, we dichotomized our variable of ground truth to positive (1) and negative (0). Feature selection was performed with k-fold cross-validation using the Elastic Net regularization method with an alpha of 0.6 and 10-fold cross-validation in MATLAB (R2019a, MathWorks, Natick, MA, USA). Feature ranking was performed to identify the individual and combined parameters best predictive for differentiating these groups. The models selected were of minimum cross-validated mean squared error. Predictive models were obtained using the top features.

Standard receiver operating characteristic (ROC) curve analysis was performed to evaluate the performance of the predictive models. ROC analysis included area under the curve (AUC), sensitivity, specificity, and accuracy. Maximization of the Youden Index was used to determine the optimal cut-off point (R2019a, MathWorks, Natick, MA, USA). A *p*-value of <0.05 was considered to indicate a statistically significant difference.

## 3. Results

*Patient and Clinical Characteristics:* Among the 21 patients with HNSCC (mean age 65 ± 12 years; age range, 47–87 years, 10 females), there were 10 with oral cavity SCC, 8 with oropharyngeal SCC, 2 with occult primary SCC with suspected head and neck origin, and 1 with parotid SCC. Of the 21 patients, 15 underwent neck dissection, and 5 underwent ultrasound-guided biopsy of suspicious lymph nodes. One patient did not have a histologic correlation, and their nodal status was determined using imaging features alone. These characteristics are displayed in Table 1.

*STIR Texture Analysis:* The results of the STIR texture analysis of benign and malignant cervical lymph nodes are summarized in Table S1. In total, 36 of 41 STIR texture features showed significant differences between benign and malignant cervical lymph nodes. Of the first-order features, 4 of 9 showed significant differences between these groups, while all 32 s-order features showed significant differences, respectively (*p* < 0.05 for all). After feature ranking, shape volume (mL), GLRLM run-length non-uniformity, GLZLM zone-length non-uniformity, and GLCM Entropy log10 were selected as independent parameters for differentiating benign and malignant lymph nodes.

Traditional FDG-PET/MR Features: There were 109 lymph nodes assessed from the pre-operative FDG-PET/MR studies in patients being evaluated for HNSCC. Among these lymph nodes, 41 were confirmed as malignant (neck dissection, *n* = 29; ultrasound-guided biopsy, *n* = 5; unequivocally metastatic, *n* = 7), while 68 were confirmed as benign. The maximum short-axis diameters were 5.3 ± 1.3 mm (range 3–10 mm) for benign lymph nodes and 10.8 ± 3.8 mm (range 5–20 mm) for malignant nodes (*p* < 0.0001). Abnormal morphology was observed in 5 of 68 benign lymph nodes, while 40 of 41 malignant lymph nodes demonstrated abnormal morphology (*p* < 0.0001). Physiologic data from FDG-PET also demonstrated significant differences between benign and malignant cervical lymph nodes. Among benign nodes, SUVmax was 3.0 ± 1.2 (range 1.4–7.3) compared to 12.6 ± 8.3 (range 4–39.4) for malignant nodes (*p* < 0.0001).

*Prediction of Malignant Nodal Disease with Texture Analysis and FDG-PET/MR Features:* The results of the ROC analysis are shown in Table 2. The best individual texture features for distinguishing these groups of lymph nodes were shape volume (mL), GLRLM run-length non-uniformity, GLZLM zone-length non-uniformity, and GLCM Entropy log10. These texture features demonstrated AUCs, sensitivity, and specificity ranging from 0.876–0.912, 65.8–82.9%, and 85.3–95.6%, respectively. Combinations of texture features improved diagnostic accuracy, with the greatest AUC, sensitivity, and specificity achieved using a combination of 22 texture features (AUC 0.952, sensitivity 92.7%, specificity 86.7%).

ROC analysis of traditional FDG-PET/MR features was also performed and is shown in Table 2. Maximum short-axis diameter differentiated these groups with an AUC of 0.935, a sensitivity of 87.8%, and a specificity of 86.7% at 7 mm. At 10 mm, sensitivity and specificity were 63.4% and 98.5% while at 15 mm, they were 17% and 100%, respectively. Lymph node morphology, classified as either normal or abnormal, yielded an AUC of 0.951, a sensitivity of 97.5%, and a specificity of 92.6%. Analysis of SUVmax was also useful for differentiating benign and malignant nodes. At SUVmax 4.6, the AUC, sensitivity, and specificity were 0.983, 97.5%, and 92.6%, respectively. There were no malignant lymph nodes with SUVmax below 4 in our dataset. SUVmax values of 3, 3.5, and 3.7 all yielded 100% sensitivity with specificity of 58.8%, 75%, and 77.9%, respectively.

MRTA of individual texture features performed comparably to lymph node size, morphology, and SUVmax alone for nodal assessment. However, a combination of 7 STIR texture features, lymph node size, morphology, and SUVmax yielded the greatest results, with an AUC, a sensitivity, and a specificity of 0.989, 97.5%, and 94.1%, respectively.

*Analysis of FDG-PET Indeterminate Nodes:* Analysis of lymph nodes with SUVmax ranging from 3.5 to 7.5 was also performed, as accurate interpretation of these lymph nodes is clinically challenging. There were 29 lymph nodes in this subset, 17 benign and 12 malignant. Among the traditional imaging features, maximum short axis diameter did not demonstrate a significant difference between these groups, while morphology and SUVmax showed significant differences. The maximum short axis diameters were 6.4 ± 1.6 mm (range 4–10 mm) for benign nodes compared to 7.3 ± 2.2 mm (range 5–12 mm) for malignant nodes (*p* = 0.2). Among the 17 benign nodes, 5 demonstrated abnormal morphology, while 11 of 12 malignant lymph nodes were considered abnormal (*p* = 0.01). SUVmax was 4.7 ± 1.2 (range 3.5–7.3) among benign lymph nodes compared to 5.6 ± 1.0 (range 4–7.4) among malignant lymph nodes (*p* = 0.04). There were no significant differences for any individual texture features between benign and malignant lymph nodes in this subset. ROC analysis demonstrated an AUC, a sensitivity, and a specificity of 0.811, 91.7%, and 70.6% for differentiating these groups using morphology alone, while the addition of 6 texture features to this assessment increased the AUC, sensitivity, and specificity to 0.912, 91.7%, and 88.2%, respectively.

**Figure 1:**
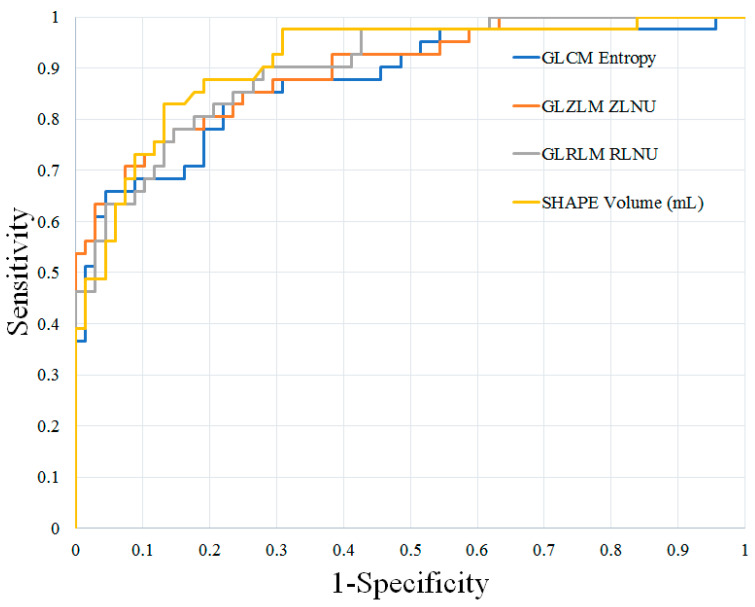
ROC curves of the best individual texture features for distinguishing benign and malignant cervical lymph nodes in patients with HNSCC. HNSCC, head and neck squamous cell carcinoma; GLCM, Gray-Level Co-Occurrence Matrix; GLZLM, Gray-Level Zone-Length Matrix; GLRLM, Gray-Level Run-Length Matrix; ZLNU, zone-length non-uniformity; RLNU, run-length non-uniformity.

**Figure 2:**
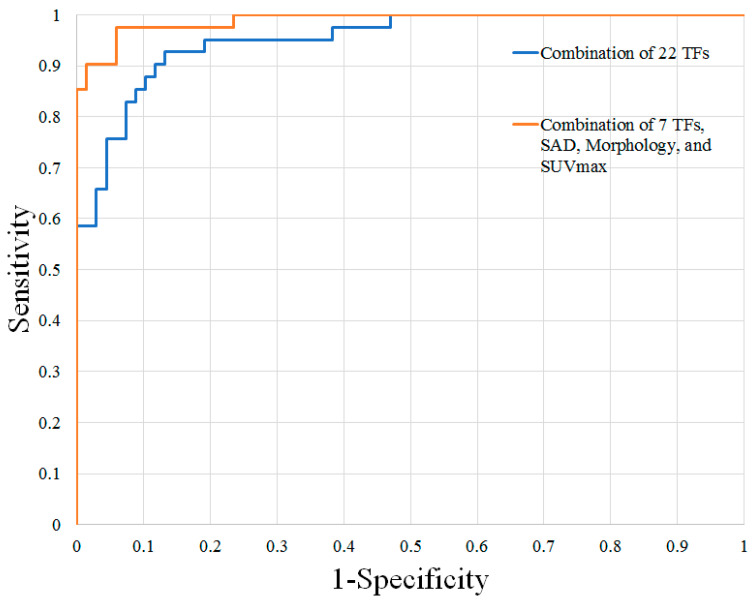
ROC curves of the top combinations for distinguishing benign and malignant cervical lymph nodes in patients with HNSCC. HNSCC, head and neck squamous cell carcinoma; TFs, texture features; SAD, short-axis diameter; SUVmax, maximum standardized uptake value.

**Figure 3:**
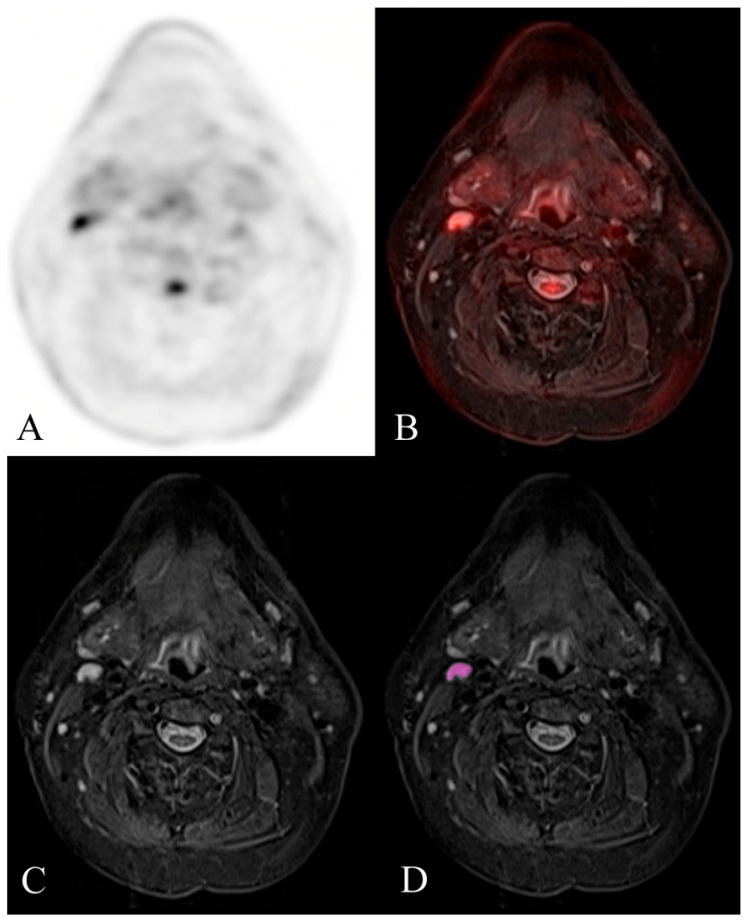
Example of a histologically confirmed benign reactive FDG-avid cervical lymph node. FDG-PET/MR evaluation of a patient with HNSCC. (**A**) attenuation-corrected FDG-PET, (**B**) fused axial STIR, (**C**) axial STIR, and (**D**) segmented axial STIR images. There is a right level 2A FDG-avid lymph node (SUVmax 6.6) that measured 7 mm in short-axis diameter and demonstrated normal morphology. Texture analysis was performed within the visualized region of interest in image (**D**). This node was histologically confirmed as benign reactive after neck dissection.

**Figure 4:**
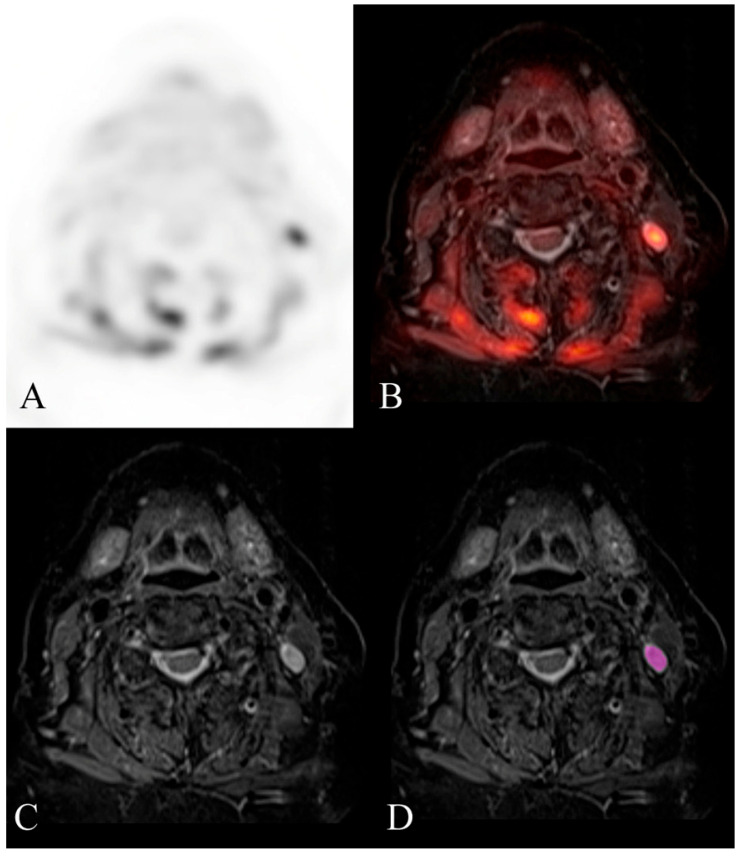
Example of a histologically confirmed malignant cervical lymph node. FDG-PET/MR evaluation of a patient with HNSCC. (**A**) attenuation-corrected FDG-PET, (**B**) fused axial STIR, (**C**) axial STIR, and (**D**) segmented axial STIR images. There is a left level 2B FDG-avid lymph node (SUVmax 9) that measured 7 mm in short-axis diameter and demonstrated abnormal morphology. Texture analysis was performed within the visualized region of interest in image (**D**). This node was histologically confirmed as malignant after neck dissection. Mild hypermetabolic activity is also visualized in the musculature of the neck, likely related to muscle strain.

The ROC curves are shown in Figure 1 and Figure 2. Examples of benign and malignant cervical lymph nodes are shown in Figure 3 and Figure 4.

## 4. Discussion

Our results demonstrate that STIR texture features derived from hybrid PET/MR technology can differentiate benign and malignant cervical lymph nodes in patients with HNSCC, with sensitivity and specificity comparable to maximum short-axis diameter, lymph node morphology, and SUVmax. Lymph node morphology and SUVmax remain accurate tools for discriminating benign and malignant nodal disease in these patients, although specificity is improved by the addition of STIR texture features, especially among FDG-PET indeterminate lymph nodes. To the best of our knowledge, the use of MRTA to improve nodal assessment specificity in HNSCC patients, especially among those with FDG-PET indeterminate nodes, has not been previously reported.

Our study adds to a large body of work showing how MRTA derived from FDG-PET/MRI can be used in conjunction with lymph node size, morphology, and SUVmax to distinguish benign and malignant lymph nodes more accurately in patients with HNSCC. With this technique, we differentiated benign and malignant cervical lymph nodes with sensitivity and specificity of 97.5% and 94.1%, which is improved compared to previously reported values of 85% and 92% using traditional FDG-PET/MR features alone [17,18]. While the addition of MRTA to lymph node morphology and SUVmax resulted in only a marginal improvement in specificity among all nodes in our study, its effect was more pronounced among FDG-PET indeterminate nodes, where specificity increased from 70.6% to 88.2% after the addition of MRTA. It is among these FDG-PET indeterminate nodes, that is, those with SUVmax ranging from 3.5 to 7.5, that we believe MRTA adds the greatest value. Reasons for this are not entirely clear, but we hypothesize that increased STIR textural coarseness among malignant nodes, resulting from increased proton richness, edema, and necrosis from tumoral infiltration of lymph node parenchyma [19,20], helped discriminate benign reactive FDG-avid nodes from truly malignant lymph nodes. Since textural coarseness has been associated with an elevated risk of recurrent disease in patients with rectal cancer and a poor prognosis among patients with ovarian cancer [21,22,23], STIR textural coarseness may also serve as a biomarker for malignant nodal disease in patients with HNSCC. 

There is disagreement in the literature regarding the optimal SUVmax threshold for distinguishing benign and malignant nodal disease among HNSCC patients undergoing PET imaging. Payabvash et al. distinguished benign and malignant cervical lymph nodes in patients with head and neck cancer with 100% sensitivity and 100% specificity at SUVmax thresholds ≥2.5 and ≥5.5 using FDG-PET/CT, respectively [24]. Nakagawa et al. found that reactive lymph nodes in oral cancer had SUVmax ranging from 1.34 to 4.53, suggesting an optimal cut-off of SUVmax 3.5 [25]. Many studies looking at quantification of FDG uptake on PET imaging for HNSCC refrain from establishing strict thresholds in part due to inherent problems associated with different scanner types, imaging time, blood glucose levels, and histological characteristics [26,27]. In our study, there were no malignant lymph nodes with SUVmax ˂ 4, while several false-positive lymph nodes had SUVmax 5.9, 6.6, and 7.3.

Our work suggests that SUVmax may be used to stratify lymph nodes as benign, indeterminate, or malignant. Based on our data, lymph nodes with SUVmax ˂ 3.5 can be classified as benign (100% sensitivity), while those with SUVmax ˃ 7.5 can be classified as malignant (100% specificity). Lymph nodes with SUVmax ranging from 3.5 to 7.5 remain indeterminate, as only 41% of nodes in this group were identified as malignant in our dataset. We found that among these FDG-PET indeterminate nodes, morphology alone and combinations of STIR texture features with or without morphology were useful for differentiating benign from malignant disease. These suggest a two-step approach utilizing FDG-PET for SUVmax and MRI for node morphology to accurately differentiate indeterminate nodal disease in patients with HNSCC. When these steps were applied to our data, there was a 94.5% diagnostic accuracy (103/109 lymph nodes correctively identified), with 5 false positives and only 1 false negative.

Prior texture analysis studies of head and neck lymph nodes have reported using ultrasound, MRI, CT, and PET-FDG (REF), with few that combined multiple methods. Kim et al. evaluated the potential role of PET/MRI for imaging metastatic lymph nodes in head and neck cancer for prediction of response to chemo-radiotherapy [17]. Safakish et al. [28] predicted head and neck cancer treatment outcomes with pre-treatment quantitative ultrasound texture features and optimized machine learning classifiers with texture-of-texture features. Zhang et al. [29] developed a pre-treatment CT-based radiomic model of lymph node response to induction chemotherapy in locally advanced HNSCC patients. Masuda et al. [30] applied machine learning to identify lymph node metastasis from thyroid cancer in patients undergoing contrast-enhanced CT studies. Yuan et al. [31] applied machine learning-based MRI texture analysis to predict occult lymph node metastasis in early-stage oral tongue squamous cell carcinoma. Baba et al. studied the pre-treatment MRI predictor of high-grade malignant parotid gland cancer using texture analysis. Sarioglu et al. [32] evaluated MRI features of parotid masses and investigated the added role of texture analysis in the differentiation of parotid tumors. Scalco et al. [21] investigated the potential of a multi-modal characterization (combination of CT, T2-weighted MRI, and diffusion-weighted MRI) at baseline and at mid-treatment, based on texture analysis, for the early prediction of LNs response to chemo-radiotherapy. In addition, deep learning analysis has also been applied to study lymph nodes in the breast [33,34,35,36]. 

The process of MRTA has inherent limitations that are relevant to our study. Firstly, due to its relatively low incidence, the small size of lymph nodes themselves presents a challenge to the process of MRTA, as second-order texture features cannot be calculated with small ROIs. Additionally, previous studies have demonstrated that texture feature values can vary with MR acquisition parameters [37]; thus, it is difficult to compare ROC analysis thresholds between studies, as is more easily accomplished with Hounsfield units or ADC values. A standardized process of ROI segmentation and analysis would improve MRTA reproducibility, potentially allowing for greater clinical utility [38]. An additional limitation was the small number of patients undergoing staging with FDG-PET/MR, as a larger sample size would increase the power of the study and address these limitations. Texture analysis and machine learning, in general, can also be applied to digital pathology slides [39,40].

## 5. Conclusions

STIR texture features derived from hybrid PET/MR technology can differentiate benign and malignant cervical lymph nodes among patients with HNSCC with accuracy comparable to lymph node size, morphology, and SUVmax. Lymph node morphology and SUVmax remain accurate tools for discriminating benign and malignant nodal disease in these patients, although specificity is improved by the addition of STIR texture features, especially among FDG-PET indeterminate lymph nodes.

## Figures and Tables

**Table 1:** Patient and Clinical Characteristics.

Characteristic	Value
Age (y)	65 ± 12 (47–87) *
No. of men	11 (52%)
Primary Mass Location:	
Oral Cavity	10 (48%)
Oral Pharynx	8 (38%)
Occult, suspected head and neck origin	2 (9%)
Parotid	1 (5%)
Tissue sampling method:	
Modified radical neck dissection	15 (71%)
Ultrasound-guided biopsy	5 (24%)

Note—data are number of patients, with percentages in parentheses. * data are mean ± standard deviation, with range in parentheses.

**Table 2:** ROC analysis of STIR texture features and traditional FDG-PET/MR features for differentiating benign and malignant cervical lymph nodes in patients with HNSCC.

Parameter	AUC	Threshold	Sensitivity (%)	Specificity (%)
GLCM Entropy log10	0.876	2.308	65.8	95.6
GLZLM ZLNU	0.898	90.25	75.6	88.2
GLRLM RLNU	0.902	150.88	78.1	85.3
Shape Volume (mL)	0.912	0.439	82.9	86.7
Combination of 22 TFs	0.952		92.7	86.7
Size (mm)	0.935	7	87.8	86.7
Morphology	0.951		97.5	92.6
SUVmax	0.983	4.6	97.5	92.6
Combination of 7 TFs, Size, Morphology, and SUVmax	0.989		97.5	94.1

Note—Threshold value is for identifying malignant nodes. 22 TFs, Histogram Skewness, Histogram Entropy log10, Histogram Entropy log2, Histogram Energy, Shape Volume (mL), Shape Sphericity, GLCM Entropy log10, GLCM Entropy log2, GLRLM SRE, GLRLM HGRE, GLRLM LRLGE, GLRLM LRHGE, GLRLM RLNU, GLRLM RP, NGLDM Contrast, NGLDM Busyness, GLZLM SZE, GLZLM LGZE, GLZLM LZLGE, GLZLM GLNU, GLZLM ZLNU, GLZLM ZP; 7 TFs, Shape Sphericity, GLCM Correlation, GLRLM SRE, GLRLM LGRE, GLRLM LRLGE, GLRLM LRHGE, NGLDM Coarseness.

## Data Availability

The data presented in this study are available on request from the corresponding author. The data are not publicly available because they contained PHI and have not yet been cleared by the IRB.

## Supplementary Materials

The following supporting information can be downloaded at: https://www.mdpi.com/article/10.3390/diagnostics14010071/s1, Table S1: Comparison of STIR texture features between all benign and malignant lymph nodes. Note—All values expressed as mean ± standard deviation. The significance threshold for difference was set at a *p* value of less than 0.05, according to an independent *t*-test. * indicates statistically significant.
