# Supplementary material for: Improved Cervical Lymph Node Characterization among Patients with Head and Neck Squamous Cell Carcinoma Using MR Texture Analysis Compared to Traditional FDG-PET/MR Features Alone
Source: Diagnostics (Basel). 2023 Dec 28;14(1):71. doi: 10.3390/diagnostics14010071 (PMC10802850; doi:10.3390/diagnostics14010071)
Supplement: Supplementary file 1 [file diagnostics-14-00071-s001.zip › diagnostics-2771039-supplementary.pdf]

**Table S1. Comparison of STIR texture features between all benign and malignant lymph nodes.** Note.–All values expressed as mean ± standard deviation. The significance threshold for difference was set at a P value of less than 0.05, according to an independent t-test. * indicates statistically significant.

| **Texture Feature** | **Benign Nodes (n=68)** | **Malignant Nodes (n=41)** | **P Value** |
|---|---|---|---|
| Histogram | | | |
| Skewness | -0.33 ± 0.43 | -0.20 ± 0.56 | .15 |
| Kurtosis | 3.08 ± 0.93 | 3.61 ± 1.11 | .0081* |
| Entropy log10 | 1.55 ± 0.05 | 1.57 ± 0.07 | 0.2 |
| Entropy log2 | 5.15 ± 0.17 | 5.20 ± 0.22 | 0.2 |
| Energy | 0.03 ± 0.005 | 0.03 ± 0.01 | 0.88 |
| Shape | | | |
| Volume (mL) | 0.30 ± 0.18 | 1.51 ± 1.82 | <.0001* |
| Volume (# vx) | 115.07 ± 66.10 | 578.71 ± 751.81 | <.0001* |
| Sphericity | 0.35 ± 0.39 | 0.48 ± 0.30 | 0.08 |
| Compacity | 0.34 ± 0.39 | 0.78 ± 0.57 | <.0001* |
| GLCM | | | |
| Homogeneity | 0.15 ± 0.04 | 0.19 ± 0.04 | <.0001* |
| Energy | 0.02 ± 0.01 | 0.01 ± 0.01 | <.0001* |
| Texture Feature | Benign Nodes (n=68) | Malignant Nodes (n=41) | P value |
| Histogram | | | |
| Skewness | -0.33 ± 0.43 | -0.20 ± 0.56 | .15 |
| Kurtosis | 3.08 ± 0.93 | 3.61 ± 1.11 | .0081* |
| Entropy log10 | 1.55 ± 0.05 | 1.57 ± 0.07 | 0.2 |
| Entropy log2 | 5.15 ± 0.17 | 5.20 ± 0.22 | 0.2 |
| Energy | 0.03 ± 0.005 | 0.03 ± 0.01 | 0.88 |
| Shape | | | |
| Volume (mL) | 0.30 ± 0.18 | 1.51 ± 1.82 | <.0001* |
| Volume (# vx) | 115.07 ± 66.10 | 578.71 ± 751.81 | <.0001* |
| Sphericity | 0.35 ± 0.39 | 0.48 ± 0.30 | 0.08 |
| Compacity | 0.34 ± 0.39 | 0.78 ± 0.57 | <.0001* |
| GLCM | | | |
| Homogeneity | 0.15 ± 0.04 | 0.19 ± 0.04 | <.0001* |
| Energy | 0.02 ± 0.01 | 0.01 ± 0.01 | <.0001* |
| Contrast | 315.18 ± 170.67 | 189.09 ± 105.12 | <.0001* |
| Correlation | 0.09 ± 0.22 | 0.23 ± 0.17 | .0007* |
| Entropy log10 | 1.93 ± 0.23 | 2.39 ± 0.31 | <.0001* |
| Entropy log2 | 6.40 ± 0.76 | 7.92 ± 1.02 | <.0001* |
| Dissimilarity | 13.62 ± 3.97 | 10.26 ± 3.08 | <.0001* |
| GLRLM | | | |
| SRE | 0.99 ± 0.01 | 0.98 ± 0.01 | <.0001* |
| LRE | 1.05 ± 0.03 | 1.09 ± 0.04 | <.0001* |
| LGRE | 0.02 ± 0.01 | 0.01 ± 0.01 | <.0001* |
| HGRE | 1501.66 ± 312.95 | 1324.09 ± 351.63 | .007* |
| SRLGE | 0.02 ± 0.01 | 0.01 ± 0.01 | <.0001* |
| SRHGE | 1480.56 ± 304.67 | 1297.78 ± 343.70 | .005* |
| LRLGE | 0.02 ± 0.01 | 0.01 ± 0.01 | <.0001* |
| LRHGE | 1589.99 ± 351.19 | 1437.97 ± 392.36 | .04* |
| GLNU | 3.74 ± 2.50 | 20.17 ± 28.57 | <.0001* |
| RLNU | 108.60 ± 58.76 | 517.99 ± 648.37 | <.0001* |
| RP | 0.98 ± 0.01 | 0.97 ± 0.01 | <.0001* |
| NGLDM | | | |
